# Vascular effects, efficacy and safety of nintedanib in patients with advanced, refractory colorectal cancer: a prospective phase I subanalysis

**DOI:** 10.1186/1471-2407-14-510

**Published:** 2014-07-11

**Authors:** Klaus Mross, Martin Büchert, Annette Frost, Michael Medinger, Peter Stopfer, Matus Studeny, Rolf Kaiser

**Affiliations:** 1 Tumor Biology Center, Department of Medical Oncology, Breisacherstrasse 117, D-79106 Freiburg in Breisgau, Germany; 2 Magnetic Resonance Development and Application Center, Department of Radiology, University Medical Center Freiburg, Freiburg, Germany; 3 Boehringer Ingelheim Pharma GmbH & Co. KG, Biberach, Germany

**Keywords:** Angiogenesis inhibitor, Clinical trial, Phase I, Nintedanib, Colorectal cancer, Magnetic resonance imaging

## Abstract

**Background:**

Nintedanib is a potent, oral angiokinase inhibitor that targets VEGF, PDGF and FGF signalling, as well as RET and Flt3. The maximum tolerated dose of nintedanib was evaluated in a phase I study of treatment-refractory patients with advanced solid tumours. In this preplanned subanalysis, the effect of nintedanib on the tumour vasculature, along with efficacy and safety, was assessed in 30 patients with colorectal cancer (CRC).

**Methods:**

Patients with advanced CRC who had failed conventional treatment, or for whom no therapy of proven efficacy existed, were treated with nintedanib ranging from 50–450 mg once-daily (n = 14) or 150–250 mg twice-daily (n = 16) for 28 days. After a 1-week rest, further courses were permitted in the absence of progression or undue toxicity. The primary objective was the effect on the tumour vasculature using dynamic contrast-enhanced magnetic resonance imaging (DCE-MRI) and expressed as the initial area under the DCE-MRI contrast agent concentration–time curve after 60 seconds (iAUC_60_) or the volume transfer constant between blood plasma and extravascular extracellular space (K^trans^).

**Results:**

Patients received a median of 4.0 courses (range: 1–13). Among 21 evaluable patients, 14 (67%) had a ≥40% reduction from baseline in K^trans^ and 13 (62%) had a ≥40% decrease from baseline in iAUC_60_, representing clinically relevant effects on tumour blood flow and permeability, respectively. A ≥40% reduction from baseline in K^trans^ was positively associated with non-progressive tumour status (Fisher’s exact: p = 0.0032). One patient achieved a partial response at 250 mg twice-daily and 24 (80%) achieved stable disease lasting ≥8 weeks. Time to tumour progression (TTP) at 4 months was 26% and median TTP was 72.5 days (95% confidence interval: 65–114). Common drug-related adverse events (AEs) included nausea (67%), vomiting (53%) and diarrhoea (40%); three patients experienced drug-related AEs ≥ grade 3. Four patients treated with nintedanib once-daily had an alanine aminotransferase and/or aspartate aminotransferase increase ≥ grade 3. No increases > grade 2 were seen in the twice-daily group.

**Conclusions:**

Nintedanib modulates tumour blood flow and permeability in patients with advanced, refractory CRC, while achieving antitumour activity and maintaining an acceptable safety profile.

## Background

Angiogenic growth factors, including vascular endothelial growth factor (VEGF), platelet-derived growth factor (PDGF) and fibroblast growth factor (FGF), and their receptors play an essential role in tumour angiogenesis [1-3]. As VEGF, acting via its endothelial receptors (VEGFR-1–3), is the most important regulator of physiological and pathological angiogenesis [1], most research into antiangiogenic therapies has focused on this signalling pathway. However, not all neoplasms respond to anti-VEGF/VEGFR agents and most, if not all, tumours that initially respond eventually develop resistance to such therapies [3]. This ‘tumour escape’, which is often observed under sustained VEGF/VEGFR inhibition, is likely to be due, at least in part, to compensatory angiogenic signalling, including that mediated by the PDGF/PDGFR and FGF/FGFR pathways [3-12]. There is also growing evidence to indicate a role for FGF and PDGF signalling in reducing the clinical efficacy of VEGF/VEGFR-targeted agents [13-15]. A role for agents with broader molecular specificity than VEGF/VEGFR alone is therefore suggested.

Lack of response and therapeutic resistance to antiangiogenic therapies is a particular problem in advanced colorectal cancer (CRC) [16,17], as exemplified by the growing number of unsuccessful phase III trials in which tyrosine kinase inhibitor/chemotherapy combinations (e.g., cediranib plus FOLFOX [5-fluorouracil, leucovorin and oxaliplatin] or CAPOX [capecitabine and oxaliplatin], vatalanib plus FOLFOX, sunitinib plus FOLFIRI [folinic acid, fluorouracil and irinotecan]) have failed to improve overall survival (OS) versus chemotherapy alone or chemotherapy combined with the anti-VEGF antibody bevacizumab (recommended as initial treatment for metastatic CRC in combination with fluoropyrimidine-based chemotherapy [18,19]) in first- or second-line use [18,20-23]. In contrast to these disappointing results, a recent phase III trial has demonstrated improved OS with the oral multikinase inhibitor regorafenib plus best supportive care (BSC) versus placebo plus BSC in patients with metastatic CRC who had progressed after failing all approved standard therapies [24]. These findings highlight the potential of angiogenesis inhibitors as salvage therapy in metastatic CRC.

Based on its broad mechanism of action (including inhibition of VEGFR 1–3, FGFR 1–3, PDGFR-α/β, RET and Flt3 [25]) and consequent potential to overcome compensatory angiogenic signalling, we explored the safety, pharmacokinetics and pharmacodynamics of the novel multi-angiokinase inhibitor nintedanib (BIBF 1120) in a phase I trial involving treatment-refractory patients with a range of advanced solid tumours [26]. As a preplanned exploratory subanalysis of this phase I study, we assessed the effect of nintedanib on the tumour vasculature in patients with heavily pretreated, advanced CRC using dynamic contrast-enhanced magnetic resonance imaging (DCE-MRI), a non-invasive imaging technique used to monitor changes in tumour haemodynamics [27]. The clinical efficacy and safety of the drug were also evaluated, as well as correlations between DCE-MRI parameters and clinical outcome. The results from this subanalysis are reported here.

## Methods

### Patients

Patients included in the phase I study were adults with advanced, non-resectable and/or metastatic, measurable solid tumours who had failed conventional treatment or for whom no therapy of proven efficacy existed; only patients with CRC were included in this subanalysis. To be enrolled, patients had to have an Eastern Cooperative Oncology Group performance status (ECOG PS) of 0 to 2, and a life expectancy of at least 3 months, and must have made a complete recovery from all prior treatment-related toxicities.

The main exclusion criteria included surgery, radiotherapy or investigational anticancer therapy (excluding nintedanib) during the previous 4 weeks; active ulcers or infectious disease; injuries with incomplete wound healing; pregnancy or breastfeeding; brain metastases requiring therapy; absolute neutrophil count <1,500/mm^3^; platelet count <100,000/mm^3^; bilirubin >1.5 mg/dL; aspartate amino transferase (AST) and/or alanine amino transferase (ALT) >3 × the upper limit of normal (or >5 × the upper limit of normal if related to liver metastases); serum creatinine >1.5 mg/dL; uncontrolled severe hypertension; and gastrointestinal disorders anticipated to interfere with the resorption of study medication.

### Study design

The phase I trial was an open-label, single and multiple dose study, with accelerated, toxicity-guided dose escalation [26]. The first treatment cycle comprised a single oral dose of nintedanib (Boehringer Ingelheim Pharma GmbH & Co. KG; administered as 50 and/or 200 mg capsules after food) on day 1, followed by a 1-day washout and 28 days of continuous once- or twice-daily oral administration of fixed-dose nintedanib. After a 1-week rest period, further cycles were permitted in the absence of major tumour progression (defined as an increase of ≥30% in the sum of the longest diameters of target lesions) or dose-limiting toxicity (DLT; defined as any drug-related toxicity ≥ Common Toxicity Criteria [CTC] grade 3, with the exception of alopecia or untreated vomiting).

The full dose-escalation protocol has been described previously [26]. Among patients with CRC, the following dose levels were evaluated: once-daily (morning) doses of 50, 100, 200, 250, 300 and 450 mg; and twice-daily (morning and evening) doses of 2 × 150, 150 + 200, 2 × 200 and 2 × 250 mg. Dose tiers were evaluated in separate patient cohorts, and intrapatient dose escalation was not permitted. Antiemetic prophylaxis was not allowed.

The primary objective of this preplanned subanalysis was to assess the effect of continuous daily dosing with nintedanib on the tumour vasculature in patients with CRC using DCE-MRI. Additional objectives included evaluation of tumour response, time to first tumour progression (TTP) and safety/tolerability.

The protocol was approved by the local medical ethics committee (Ethik-Kommission der Albert-Ludwigs-Universität Freiburg), and the trial was conducted in accordance with the Declaration of Helsinki and Good Clinical Practice guidelines. All patients provided written informed consent prior to engaging in study procedures.

### Assessments

#### ***Dynamic contrast-enhanced magnetic resonance imaging***

Full details of the DCE-MRI protocol that was used have been published previously [28,29]. In brief, coronal slice images through one or more measurable, clearly defined, non-necrotic target lesions were obtained at baseline (screening), on day 2 for once-daily dosing or day 3 for twice-daily dosing, and on day 29/30 of the first treatment cycle immediately prior to and following intravenous administration of contrast agent (low-molecular weight gadolinium-DTPA) via a standard power injector. Additional images were obtained on day 28 of each repeated cycle for all patients remaining in the trial.

All imaging data were acquired using a clinical 1.5-Tesla whole-body magnetic resonance system (Sonata, Siemens, Germany) applying the T1-weighted inversion recovery TrueFISP pulse sequence, an approach that offers high temporal resolution and accuracy at least as good as the widely used 3D-Flash protocol [29,30]. The data obtained from the scans were used to determine the change in contrast agent concentration in tumour tissue over time.

For this analysis, the two endpoints of interest were (1) the initial area under the contrast agent concentration–time curve for the initial 60 seconds after onset of contrast agent uptake (iAUC_60_); and (2) the transfer constant for the transfer of contrast agent from inside tumour blood vessels to the extravascular-extracellular space (K^trans^). Both parameters, which are influenced by blood flow and vascular permeability properties of the tumour, were calculated from the imaging data using standard methods [31,32].

#### ***Tumour assessment***

Target tumour lesions were assessed by computed tomography or MRI according to *Response Evaluation Criteria in Solid Tumors* (RECIST) version 1.0 [33]. Tumour evaluations were undertaken at baseline and at the end of each treatment cycle.

#### ***Safety and tolerability***

The safety and tolerability of nintedanib were assessed by adverse event (AE) reporting, physical examination, vital signs, 12-lead resting electrocardiogram and laboratory safety parameters. AEs were recorded at each scheduled visit and graded according to CTC version 2.0. Safety laboratory parameters (haematology, coagulation parameters, clinical chemistry, tumour markers and urinalysis) were assessed at regular intervals throughout the study.

### Statistical analyses

Analyses were restricted to CRC patients who had received at least one dose of nintedanib and for whom data at and/or after baseline were available. For the DCE-MRI analysis, the proportion of evaluable patients (i.e., those with measurable, non-necrotic target tumour lesions) with a ≥40% reduction from baseline in tumour K^trans^ or iAUC_60_ was determined, as this represents the threshold for a clinically relevant antivascular response [34]. Logistic regression models were fitted with DCE-MRI response parameters (<40% vs. ≥40% reduction from baseline in K^trans^ or iAUC_60_) as explanatory variables and clinical outcome (complete or partial response, or stable disease vs. disease progression) as the dependent variable. Two-sided Fisher’s exact tests were then used to investigate contingencies (i.e., the generic correlation) between DCE-MRI responses and clinical outcome. p-values of <0.05 were reported as nominally significant.

Tumour responses and safety variables were analysed using descriptive statistics, and TTP (defined as the time elapsed from first administration of study medication to tumour progression) was estimated using Kaplan-Meier methodology. A log-rank test was used to compare the Kaplan-Meier curves for TTP between the two dosing schedules (once- vs. twice-daily) of nintedanib.

## Results

### Patients

A total of 30 patients with advanced, non-resectable and/or metastatic CRC were treated with increasing doses of nintedanib once- (n = 14) or twice- (n = 16) daily at a single centre in Germany between November 2002 and November 2004. The demographics and baseline characteristics of patients within this highly treatment-refractory CRC subgroup are shown in Table 1. Although most baseline parameters were well balanced, there were some quantitative differences between the two dosing groups (once- vs. twice-daily) in terms of sex, time since diagnosis, clinical stage at diagnosis and lung metastases. All patients had metastatic CRC (≥1 metastatic site) and had received 1–5 lines of chemotherapy during the metastatic stage. No patient had received bevacizumab or cetuximab prior to study inclusion; one patient had received sorafenib which at the time of the study was considered an RAF kinase inhibitor rather than a multikinase angiogenesis inhibitor. One patient had previously received adjuvant chemo-radiotherapy and was included in the study after rejecting all standard treatments. The patient was subject to two dose reductions and subsequently excluded from the study due to DLT.

**Table 1:** Patient demographics and baseline characteristics

**Parameters**	**Nintedanib once-daily (n = 14)**	**Nintedanib twice-daily (n = 16)**
Median age, years (range)	58.0 (41–74)	59.5 (34–74)
Sex, n (%)		
Male	9 (64)	15 (94)
Female	5 (36)	1 (6)
ECOG performance status, n (%)		
0	2 (14)	5 (31)
1	10 (71)	10 (63)
2	1 (7)	1 (6)
Unknown	1 (7)	0
Median time since diagnosis, days (range)	733 (325–2,214)	1,006 (229–2,968)
Prior treatment for CRC, n (%)		
Chemotherapy	14 (100)	16 (100)
Radiotherapy	4 (29)	5 (31)
Surgery	14 (100)	16 (100)
Immunotherapy	1^a^ (7)	0
Hormone therapy	1 (7)	1 (6)
Clinical stage at diagnosis, n (%)		
Stage I	0	2 (13)
Stage II	1 (7)	1 (6)
Stage III	1 (7)	7 (44)
Stage IV	12 (86)	6 (38)
Location of metastatic sites,^b^ n (%)		
Lung	5 (38)	9 (56)
Liver	10 (71)	11 (69)
Lymph nodes	6 (43)	7 (44)
Bone	1 (7)	0
Median number of metastatic sites, n (range)	2 (1–4)	2 (1–5)

Percentages may not add up to 100% due to rounding. ^a^Patient received panorex; ^b^Not all metastatic sites are listed. Abbreviations: CRC = colorectal cancer, ECOG = Eastern Cooperative Oncology Group.

Patients on the once-daily schedule of nintedanib received doses of between 50 and 450 mg once-daily, while those on the twice-daily schedule received doses of between 150 (total dose 300 mg/day) and 250 (total dose 500 mg/day) mg twice-daily (Table 2). Overall, patients were treated for a median of 4.0 cycles (range: 1–13 cycles) with 15 of the 30 patients (50%) receiving >2 cycles. Of the 30 patients who were enrolled, 15 (50%) continued study treatment until disease progression.

**Table 2:** Patient exposure to nintedanib

**Dose (once-daily)**	**Patients (n)**	**Dose (twice-daily)**	**Patients (n)**
50 mg	1	150 mg × 2	4
100 mg	1	150 mg + 200 mg	4
200 mg	4	200 mg × 2	1
250 mg	4	250 mg × 2	7
300 mg	2	–	–
450 mg	2	–	–

### Dynamic contrast-enhanced magnetic resonance imaging

Twenty-one patients with CRC were evaluable for DCE-MRI. In total, 14 of the 21 patients with evaluable DCE-MRI data (67%) had a ≥40% reduction from baseline in tumour K^trans^, representing a clinically relevant antivascular effect [34]. Similarly, 13 of the 21 patients (62%) had a ≥40% decrease from baseline in tumour iAUC_60_.

In the correlative analyses, a ≥40% reduction from baseline in K^trans^ was shown to be positively associated with non-progressive tumour status (complete or partial response, or stable disease; Fisher’s exact test: p = 0.0032).

**Figure 1:**
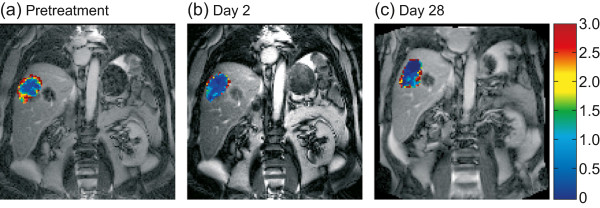
**K**^**trans **^**maps from a patient with liver metastases treated with nintedanib 250 mg once-daily (#18).** Maps were registered to original TrueFISP images taken **(a)** pretreatment, **(b)** on day 2 and **(c)** on day 28. Abbreviation: K^trans^ = volume transfer constant between blood plasma and extravascular extracellular space.

**Figure 2:**
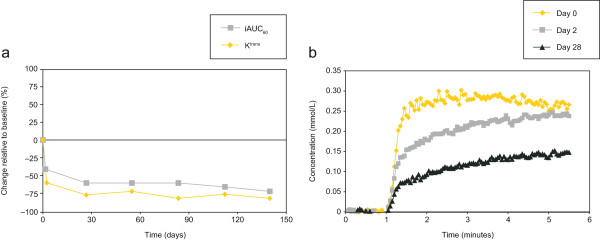
**DCE-MRI parameters in a patient with liver metastases treated with nintedanib 250 mg once-daily (#18). (a)** Change in K^trans^ and iAUC_60_ from baseline over time; and **(b)** concentration–time curves for contrast agent averaged over the whole region of interest at baseline, day 2 and day 28. Both figures illustrate a strong reduction of contrast agent uptake in the target tumour metastasis on day 2 and on subsequent assessments. Abbreviations: DCE-MRI = dynamic contrast-enhanced magnetic resonance imaging, K^trans^ = volume transfer constant between blood plasma and extravascular extracellular space, iAUC_60_ = initial area under the DCE-MRI contrast agent concentration–time curve after 60 seconds.

Figure 1 shows parameter maps of K^trans^, taken pretreatment, and on days 2 and 28, from a patient with liver metastases who received nintedanib 250 mg once-daily. As shown in Figure 2a, K^trans^ and iAUC_60_ decreased relative to baseline over time in this patient who had stable disease according to RECIST. A strong reduction in contrast agent uptake was observed relative to baseline in the target tumour lesion from this patient on both day 2 and day 28 (Figure 2b).

### Efficacy

**Figure 3:**
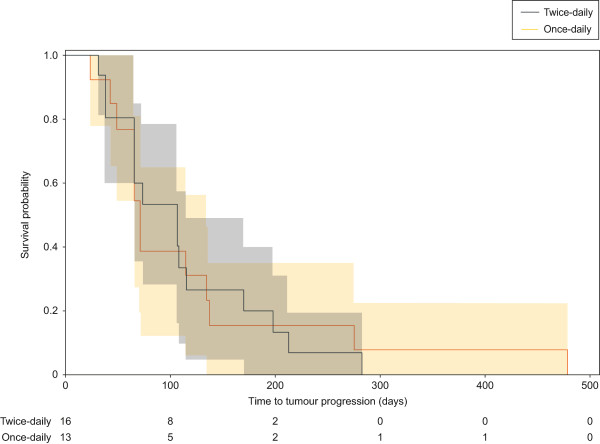
**Kaplan-Meier plot showing time to first tumour progression by nintedanib dosing schedule.** The shaded areas represent 95% confidence intervals. Abbreviations: BID = twice-daily, QD = once-daily.

One patient (3%) with CRC and liver metastasis who was treated with nintedanib 250 mg twice-daily achieved a partial response, while 24 patients (80%) treated with either schedule at various dose levels had a best response of stable disease lasting ≥8 weeks.Based on Kaplan-Meier estimates (including data from patients who rolled over to an extension study, but excluding data from one patient in which TTP was censored, and censoring time was not available), median TTP was 71 days (95% confidence interval [CI]: 48–134 days) among patients who received once-daily nintedanib and 106 days (95% CI: 37–115 days) among patients who received the twice-daily schedule (Figure 3). The difference between the two dosing schedules was not statistically significant (hazard ratio [HR]: 1.036 [95% CI: 0.842–2.225]; log-rank test: p = 0.9274). Among all evaluable patients with CRC, the 4-month TTP rate was 26% (95% CI: 17–43%) and median TTP was 72.5 days (95% CI: 65–114 days).

### Safety and tolerability

The most frequent drug-related AEs reported across all treatment cycles and dose levels/schedules were nausea, vomiting and diarrhoea (Table 3). The majority of drug-related AEs were CTC grade 1 or 2 in intensity, including all gastrointestinal AEs (Table 3), and mostly occurred during the first treatment cycle independently of the dosing schedule (data not shown). Drug-related AEs ≥ CTC grade 3 were only seen in three patients, all of whom had received the twice-daily schedule of nintedanib. Two patients experienced CTC grade 1 drug-related hypertension. No treatment-related deaths were reported.

**Table 3:** Summary of nintedanib-related toxicities

	**CTC grade, n (%)**				
**Patients with drug-related AEs, n (%)**	**1**	**2**	**3**	**4**	**Total**
Total	18 (60)	6 (20)	2 (7)	1 (3)	27 (90)
Gastrointestinal disorders	16 (53)	7 (23)	0	0	23 (77)
Nausea	16 (53)	4 (13)	0	0	20 (67)
Vomiting	14 (47)	2 (7)	0	0	16 (53)
Diarrhoea	7 (23)	5 (17)	0	0	12 (40)
Investigations	2 (7)	1 (3)	2 (7)	1 (3)	6 (20)
Hepatic enzyme increased	0	1 (3)	0	1 (3)	2 (7)
ALT increased	0	1 (3)	0	0	1 (3)
AST increased	0	1 (3)	1 (3)	0	2 (7)
GGT increased	0	1 (3)	0	0	1 (3)
CD4 decreased	0	0	2 (7)	0	2 (7)
General disorders	5 (17)	0	0	0	5 (17)
Fatigue	5 (17)	0	0	0	5 (17)

*Abbreviations*: *AE* adverse event, *ALT* alanine aminotransferase, *AST* aspartate aminotransferase, *CTC* Common Toxicity Criteria, *GGT* gamma-glutamyl transpeptidase.

Four of the 14 patients treated with once-daily nintedanib experienced an increase in ALT and/or AST ≥ CTC grade 3. In contrast, there were no ALT/AST increases > CTC grade 2 in the 16 patients receiving twice-daily nintedanib. Most increases in hepatic enzymes reported during twice-daily dosing were seen after the first treatment cycle. No treatment-related elevations in bilirubin or alkaline phosphatase were observed in either dosing group.

## Discussion

While the injectable anti-VEGF monoclonal antibody bevacizumab is a well-established first-/second-line treatment option for advanced CRC [18,19], trials of oral, small molecule antiangiogenic agents have been largely unsuccessful in this setting. To date, the only oral antiangiogenic therapy to have succeeded in a phase III trial in advanced CRC is regorafenib, a multikinase inhibitor of VEGFR 1–3, TIE2, PDGFR-β, FGFR-1, c-KIT, RET and B-RAF [24,35,36]. In this phase III trial, regorafenib plus BSC significantly increased median OS by 1.4 months compared with placebo plus BSC (6.4 vs. 5.0 months, respectively; HR: 0.77 [95% CI: 0.64–0.94]; p = 0.0052) in patients who had progressed after all standard therapies. These positive results indicate a role for small molecule antiangiogenic therapies in the treatment of advanced CRC, at least in the salvage setting.

In our prospective subanalysis of a phase I trial [26], DCE-MRI was used to investigate the effects of the oral angiokinase inhibitor nintedanib (administered once- or twice-daily) on tumour blood perfusion and vascular permeability in 30 patients with heavily pretreated, advanced, non-resectable and/or metastatic CRC--that is, characteristics akin to those seen in patients enrolled in the regorafenib phase III trial [24]. DCE-MRI utilises a low-molecular weight paramagnetic contrast agent (in this case gadolinium-DTPA) that diffuses readily from the tumour blood supply to the extravascular extracellular space. On acquisition of rapid images, the time course of the signal intensity change induced by the contrast agent, which directly reflects its intra- and extravascular concentration in the tumour region of interest, may be followed.

The results of our analysis showed that, like many other angiogenesis inhibitors [37-45], nintedanib can exert clinically meaningful antiangiogenic effects on the tumour vasculature (in >60% of evaluable patients), as defined by ≥40% reductions from baseline in iAUC_60_ and K^trans^ [34]. The strong antivascular effect seen with nintedanib may result from its potential to simultaneously inhibit multiple angiogenic and mitogenic signalling pathways (mediated by VEGFR, PDGFR, FGFR, RET and Flt3 [25]), which may enable the drug to block compensatory angiogenic pathways that can be activated when anti-VEGF agents are used in isolation [3-12].

Despite some inter-patient variability in DCE-MRI parameters, a ≥40% reduction from baseline in K^trans^ was shown to be positively associated with non-progressive tumour status (p = 0.0032). This finding suggests that DCE-MRI K^trans^ response may be a potential marker of disease control during nintedanib treatment. Importantly, the results mirror those in the overall phase I population and support other data suggesting DCE-MRI as a potentially useful surrogate marker for defining the pharmacological response to angiogenesis inhibitors in CRC [26,34,46,47].

In the RECIST analysis of tumour response, one patient achieved a partial response and a further 24 achieved stable disease lasting for ≥8 weeks, resulting in a disease control rate of 83%, 4-month TTP rate of 26% and median TTP of 72.5 days. These efficacy data are very similar to those obtained with regorafenib in the aforementioned phase III study of 760 patients with metastatic CRC who had failed all standard therapies [24]. In the phase III trial, 4-month progression-free survival (PFS) was 20% in the regorafenib plus BSC arm and 4% in the placebo plus BSC arm. The data are also comparable to those seen in an earlier phase I dose-escalation, monotherapy study of regorafenib in 53 patients with treatment-refractory advanced solid tumours, where a disease control rate of 66% was reported [48]. Among 38 patients with heavily pretreated advanced CRC (median 4 prior lines of therapy), who were enrolled in an expansion cohort to this regorafenib phase I trial, the disease control rate was 74% and median PFS was 107 days [36]. Although further studies are clearly needed, the similarity of the TTP/PFS data and patient populations between the regorafenib trials and the present subanalysis implies that nintedanib may be potentially active in the salvage setting.

The activity of nintedanib in CRC is further supported by recent data demonstrating similar efficacy and improved tolerability of nintedanib plus modified FOLFOX6 versus bevacizumab plus mFOLFOX6 in a randomised phase II study of 126 patients with previously untreated metastatic CRC [21]. In the phase II trial, 9-month PFS was shown to be 63% (95% CI: 50–75%) in the nintedanib plus mFOLFOX6 arm versus 69% (95% CI: 53–86%) in the bevacizumab plus mFOLFOX6 arm, while median PFS was 10.6 months (95% CI: 9.4–12.3 for nintedanib/mFOLFOX6 and 9.1–not reached for bevacizumab/mFOLFOX6) in both arms. The objective response rate was 61% and 54%, respectively. In terms of safety, nintedanib plus mFOLFOX6 was associated with lower incidences of serious AEs (34% vs. 54%) and serious gastrointestinal AEs (12% vs. 29%) than bevacizumab plus mFOLFOX6, indicating improved tolerability of the nintedanib-containing regimen [21].

Reassuringly, the safety profile of nintedanib observed in the present study was entirely consistent with that seen in other monotherapy studies conducted in patients with a range of solid tumours, including CRC [26,49-52]. Nintedanib doses of up to 500 mg/day were generally well tolerated with no reports of new or unexpected toxicities. The most common drug-related toxicities were mild or moderate gastrointestinal AEs (nausea, vomiting and diarrhoea) and mild or moderate, reversible hepatic enzyme elevations. Most gastrointestinal AEs occurred during the first treatment cycle and responded well to medical intervention. Furthermore, all hepatic enzyme increases responded quickly (within 2 weeks) to treatment interruption/discontinuation or dose reduction. Unlike other angiogenesis inhibitors, such as regorafenib, pazopanib, sorafenib or sunitinib [24,36,48,53-56], nintedanib was not associated with skin toxicity, and reports of hypertension (n = 2, both CTC grade 1) were uncommon; these findings suggest a favourable comparative safety profile for nintedanib.

In terms of limitations, this subanalysis is clearly constrained by the non-randomised design of the phase I study and limited sample size. Nevertheless, analyses such as these are useful for hypothesis generation, and some of the interesting findings reported here warrant further investigation.

## Conclusions

DCE-MRI assessments of iAUC_60_ and K^trans^ responses provide evidence that the multi-angiokinase inhibitor nintedanib can modulate tumour blood flow and permeability in patients with advanced, refractory CRC, while maintaining an acceptable, manageable safety profile. A RECIST response of stable disease or better was also observed in >80% of this population of heavily pretreated patients; encouraging results that support further clinical investigation of nintedanib in this salvage setting.

## Abbreviations

AE: Adverse event; ALT: Alanine amino transferase; AST: Aspartate amino transferase; BSC: Best supportive care; CRC: Colorectal cancer; CTC: Common toxicity criteria; DCE-MRI: Dynamic contrast-enhanced magnetic resonance imaging; ECOG PS: Eastern Cooperative Oncology Group performance status; FGF: Fibroblast growth factor; FGFR: FGF receptor; HR: Hazard ratio; iAUC_60_: Initial area under the DCE-MRI contrast agent concentration–time curve after 60 seconds; K^trans^: Volume transfer constant between blood plasma and extravascular extracellular space; OS: Overall survival; PDGF: Platelet-derived growth factor; PDGFR: PDGF receptor; PFS: Progression-free survival; RECIST: Response evaluation criteria in solid tumors; TTP: Time to first tumour progression; VEGF: Vascular endothelial growth factor; VEGFR: VEGF receptor.

## Competing interests

PS, MS and RK are employees of Boehringer Ingelheim; KM and MB have received research funding from Boehringer Ingelheim (funding for MB to perform the magnetic resonance imaging was received via KM); AF and MM have no competing interests to declare.

## Authors’ contributions

KM, AF and MM recruited patients, as well as collected and analysed the data. MB carried out the magnetic resonance imaging and analysis. KM, PS, MS and RK were involved in study design and data analysis. All authors were fully responsible for all content and editorial decisions, were involved at all stages of manuscript development, and have approved the final version.

## Pre-publication history

The pre-publication history for this paper can be accessed here:

http://www.biomedcentral.com/1471-2407/14/510/prepub
